# Perceptions of COVID-19 vaccine side effects by political affiliation

**DOI:** 10.1093/pubmed/fdad105

**Published:** 2023-07-06

**Authors:** David Farabee, Angela Hawken

**Affiliations:** Department of Population Health, New York University Grossman School of Medicine, New York, NY, USA; New York University Marron Institute, New York, NY, USA

**Keywords:** bias, COVID-19, perceptions, SARS-CoV-2, side effects, vaccine

## Abstract

**Background:**

We sought to assess the extent to which subjective experiences of COVID-19 vaccine side effects among US adults are associated with political party identification.

**Methods:**

An online survey was conducted of a national sample of US adults (*N* = 1259) identifying as either Republican or Democrat.

**Results:**

There was no significant difference by party identification in the perceived severity of vaccination side effects; however, Republicans were significantly less likely to recommend the vaccine to others in light of their experience (OR = 0.40; 95% CI, 0.31–0.51; *P* < 0.001). Republicans also reported having a larger share of COVID-19-vaccinated friends and family who experienced notable side effects (OR = 1.31; 95% CI, 1.02–1.68; *P* < 0.05). There was a positive association between respondents’ perceived side-effect severity and the proportion of peers who also reported notable side effects (*r* = 0.43; *P* < 0.001).

**Conclusion:**

Subjective appraisals of the vaccinated may affect broader vaccine acceptability.

## Background

It is estimated that COVID-19 vaccine programs reduced COVID-19-related deaths by nearly two-thirds globally, saving as many as 19.8 million lives in the 1st year of their availability (8 December 2020–2021).^1^ However, the public health benefits of COVID-19 vaccines were undermined in countries where hesitancy rates were high, including in the United States during the early phase of the vaccine rollout.^2^

Among adults in the United States, the likelihood of receiving a COVID-19 vaccine is strongly associated with political affiliation, with Republicans significantly less likely than Democrats to be vaccinated.^3^^,^^4^ Less understood is the role that political affiliation may play in the perceived side effects of the vaccine ‘among those who are vaccinated’. This has important implications, as the experiences of those who are vaccinated may carry heightened influence within a vaccine recipient’s social network, and many vaccine-hesitant adults consider friends and family to be the most trustworthy sources of information.^5^

To better understand the relationship between political affiliation and COVID-19 vaccine experiences, data were analyzed from a national survey of American adults who identify as either Democrat or Republican, to assess whether political affiliation is associated with (i) subjective appraisals of severity of side effects, (ii) the role that these experiences play in encouraging (or discouraging) others in the respondents’ social network to receive a COVID-19 vaccine and (iii) the vaccine experience of other members of the respondents’ social network.

## Methods

Data for this study were collected from an online survey panel by Centiment, LLC, a market-research firm, and provided to the researchers fully anonymized. Respondents were directly recruited, from across the United States, who identified as Republican or Democrat with directional balancing on age, gender and census region. The NYU Grossman School of Medicine Institutional Review Board considered this study not human subjects research because of the lack of interaction with or collection of identifiable information about human subjects.

Of the 1383 respondents who entered the survey, 1259 (91%) passed the screening criterion (identifying as a Republican or Democrat) and completed the survey. Differences in baseline characteristics and responses were examined with a *t*-test (quantitative data) and *χ*^2^ test (dichotomous variables). Ordered logistic regressions were used to study differences in ordinal outcomes, controlling for other factors.

## Results

The sample includes similar numbers of Republicans (*n* = 629) and Democrats (*n* = 630). Republicans were older (48 [SD = 17.1 versus 43 [SD = 17.1], *P* < 0.0001), more likely to be male (57 versus 47%, *P* < 0.0001), and more likely to identify as White (85.2 versus 58.1, *P* < 0.001). Consistent with prior research, Democrats were more likely than their Republican counterparts to report having received at least one COVID-19 vaccine (84 versus 59%, respectively; *P* < 0.001), and to have received a higher number of doses (mean [SD], 2.4 [1.4] versus 1.5 [1.5]; *P* < 0.001). Among the vaccinated, Republicans reported having experienced significant side effects similarly to Democrats (21 versus 25%, respectively; *P* = 0.17; see Table 1).

**Table 1:** Sample characteristics by political affiliation (*N* = 1259)

	Republican	Democrat	Total
Sample size	*n* = 629	*n* = 630	*n* = 1259
Demographics			
% Female^*^^*^	42.9%	56.0%	49.5%
Mean age [SD]^*^^*^	48 [17.1]	43 [17.1]	46 [17.3]
Race and ethnicity			
Black, non-Hispanic^*^^*^	3.5%	24.9%	14.2%
Hispanic^*^^*^	8.1%	14.3%	11.2%
White, non-Hispanic^*^	85.2%	58.1%	71.6%
Other	3.2%	2.7%	2.9%
Vaccine report			
% receiving at least one vaccine dose^*^^*^	59.1%	84.4%	71.8%
Mean # of doses^*^^*^	1.51 [1.48]	2.41 [1.43]	2.7 [1.1]
% reporting experiencing notable side effects	21.2%	25.2%	23.6%

^^*^^ *P* < 0.01;

^^*^^*^^ *P* < 0.001

**Fig. 1:**
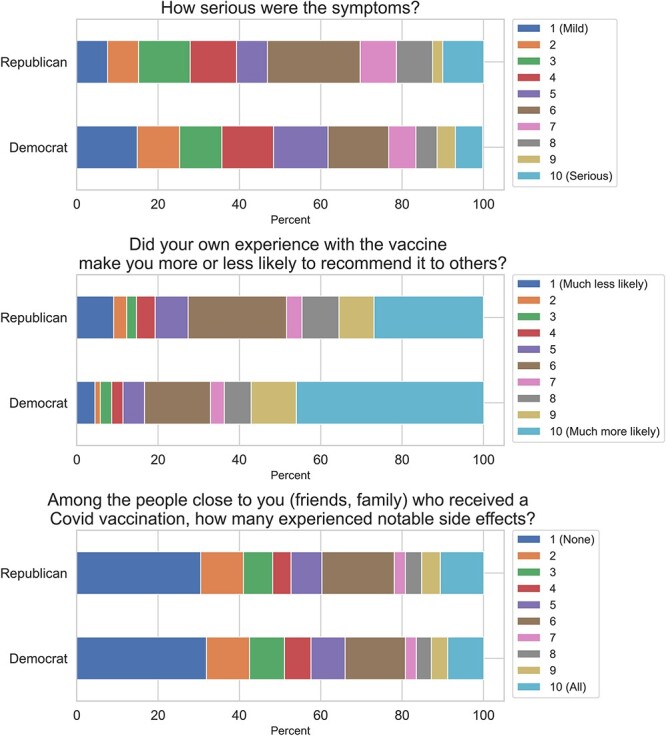
Distribution of responses on Primary-Outcome Survey Questions.

Table 2 presents ordered-logistic-regression results of (i) the perceived severity of side effects, (ii) the extent to which the respondent’s experience with the vaccine led them to be more or less likely to recommend it to others in their social network and (iii) the vaccine experience of people in the respondents’ social network, controlling for gender, age and race (the raw distribution of responses to these survey items is presented in Fig. 1). Among those reporting symptoms, there was no significant difference in Republicans’ rating of the severity of their side effects (OR = 1.38; 95% CI, 0.82–2.3). However, Republicans (relative to Democrats) were significantly less likely to recommend the vaccine to others in light of their experience (OR = 0.40; 95% CI, 0.31–0.51; *P* < 0.001), and Republicans (relative to Democrats) reported having a larger share of COVID-19-vaccinated friends and family who experienced notable side effects (OR = 1.26; 95% CI, 1.02–1.55; *P* < 0.05). There was a positive correlation between respondents’ perceived side-effect severity and the proportion of friends and family who also reported notable side effects (*r* = 0.43; *P* < 0.001).

**Table 2:** Predictors of COVID-19 vaccine perceptions (*N* = 1259)

Characteristic	Side-effect severity (n = 213) Odds ratio (95% CI)	Recommend to others (n = 904) Odds ratio (95% CI)	Experience of social network (n = 1259) Odds ratio (95% CI)
Female	0.85 (0.52–1.38)	1.03 (0.81–1.31)	1.03 (0.84–1.13)
Age	1.00 (0.99–1.02)	1.01 (1.00–1.02)^*^^*^	0.98 (0.97–0.98)^*^^*^^*^
Republican	1.38 (0.83–2.32)	0.40 (0.31–0.51)^*^^*^^*^	1.26 (1.02–1.55)^*^
Race/ethnicity (Ref White/non-Hispanic)			
-Black, non-Hispanic	0.65 (0.31–1.37)	0.75 (0.52–1.07)	0.84 (0.62–1.13)
-Hispanic	0.87 (0.45–1.70)	0.82 (0.55–1.23)	1.01 (0.74–1.41)
-Other	1.26 (0.26–5.99)	1.07 (0.55–2.07)	0.93 (0.50–1.75)

^^*^^ *P* < 0.05;

^^*^^*^^ *P* < 0.01;

^^*^^*^^*^^ *P* < 0.001

## Discussion

### Main finding of this study

Results of this survey replicate the finding that political party affiliation in the United States is associated with the likelihood of receiving a COVID-19 vaccine. Among those who were vaccinated, the subjective appraisals of side effects were similar across both groups. But these similar experiences were associated with divergent reactions, with Republicans more likely to discourage others from becoming vaccinated themselves. Furthermore, Republicans reported having a higher proportion of friends and family who experienced negative side effects from the vaccine, which correlated with their own perceptions of side-effect severity.

Whether these findings evince a negative bias among Republicans or a positive bias among Democrats cannot be established with this survey. But two clear patterns emerge from these data: (i) political party identification in the United States appears to modulate COVID-19 vaccine perceptions even among those who receive the vaccine and (ii) individual perceptions of side-effect severity are positively correlated with those of one’s social network. As a result, the subjective appraisals of the vaccinated merit further attention, as they are likely to affect broader vaccine acceptability within their distinct social networks.^6^

### What is already known on this topic

As indicated in the introduction to this paper, it is well established that the USA suffered higher rates of COVID-19-related mortality than its industrialized counterparts, and that this was largely related to vaccine hesitancy. A recent analysis found that Republican Party affiliation was the strongest single predictor of COVID-19 vaccine refusal.^7^ Likewise, a recent analysis by Bilinski *et al*.^8^ found that COVID-19-related deaths in the USA were significantly higher than that among peer countries, though this disparity was ‘muted’ among the 10 US states with the highest vaccination rates.

### What this study adds

This study contributes new information to the role of political affiliation and vaccine acceptance by focusing on the perceived side effects of the vaccine among those who are vaccinated. This is an important group, as those who receive a vaccination are generally considered to be more credible messengers of its consequences than those who do not. Although a considerable amount of research has centered on the decision to get vaccinated, this study demonstrated that political affiliation continued to shape perceptions of the vaccine even among the subset of the population who followed public heath recommendations to receive it. The persistence of these perceptual differences among the vaccinated population is an important of focus for future public health messaging because, as we have also shown, there is a significant association between an individual’s perceived vaccine side effects and those of that individual’s social network.

### Limitations of this study

The primary limitations of this study are that the data are based on self-reports, and that the sample was collected though an online survey. Although clinical records regarding vaccine participation and adverse events would have strengthened this study, our focus on side-effect perceptions required a reliance on self-reports. With regard to the sample frame of online survey participants, such an approach excludes those without access to the internet, such as those who unhoused or incarcerated. Omission of incarcerated adults is of particular concern, given the higher levels of vaccine hesitancy among the carceral population.^9^

## Conflict of interest

DF has received in-kind study medication from Alkermes and Indivior. AH reports no conflicts of interest. The authors alone are responsible for the content and writing of this paper.

## Funding

This research was self-funded.

## Data availability

The data underlying this article will be shared on reasonable request to the corresponding author.

## Notes

**David Farabee**, Research Professor

**Angela Hawken**, Professor
